# Contextual blending of ingroup/outgroup face stimuli and word valence: LPP modulation and convergence of measures

**DOI:** 10.1186/1471-2202-10-69

**Published:** 2009-06-26

**Authors:** Esteban Hurtado, Andrés Haye, Ramiro González, Facundo Manes, Agustiń Ibáñez

**Affiliations:** 1 Laboratory of Cognitive Neuroscience, Universidad Diego Portales, Santiago, Chile; 2 Researcher Career, National Scientific and Technical Research Council (CONICET), Buenos Aires, Argentina; 3 Laboratory of Experimental Psychology and Neuroscience, Institute of Cognitive Neurology (INECO), Buenos Aires, Argentina; 4 School of Engineering, Pontificia Universidad Católica de Chile, Santiago, Chile; 5 School of Psychology, Pontificia Universidad Católica de Chile, Santiago, Chile

## Abstract

**Background:**

Several event related potential (ERP) studies have investigated the time course of different aspects of evaluative processing in social bias research. Various reports suggest that the late positive potential (LPP) is modulated by basic evaluative processes, and some reports suggest that in-/outgroup relative position affects ERP responses. In order to study possible LPP blending between facial race processing and semantic valence (positive or negative words), we recorded ERPs while indigenous and non-indigenous participants who were matched by age and gender performed an implicit association test (IAT). The task involved categorizing faces (ingroup and outgroup) and words (positive and negative). Since our paradigm implies an evaluative task with positive and negative valence association, a frontal distribution of LPPs similar to that found in previous reports was expected. At the same time, we predicted that LPP valence lateralization would be modulated not only by positive/negative associations but also by particular combinations of valence, face stimuli and participant relative position.

**Results:**

Results showed that, during an IAT, indigenous participants with greater behavioral ingroup bias displayed a frontal LPP that was modulated in terms of complex contextual associations involving ethnic group and valence. The LPP was lateralized to the right for negative valence stimuli and to the left for positive valence stimuli. This valence lateralization was influenced by the combination of valence and membership type relevant to compatibility with prejudice toward a minority. Behavioral data from the IAT and an explicit attitudes questionnaire were used to clarify this finding and showed that ingroup bias plays an important role. Both ingroup favoritism and indigenous/non-indigenous differences were consistently present in the data.

**Conclusion:**

Our results suggest that frontal LPP is elicited by contextual blending of evaluative judgments of in-/outgroup information and positive vs. negative valence association and confirm recent research relating in-/outgroup ERP modulation and frontal LPP. LPP modulation may cohere with implicit measures of attitudes. The convergence of measures that were observed supports the idea that racial and valence evaluations are strongly influenced by context. This result adds to a growing set of evidence concerning contextual sensitivity of different measures of prejudice.

## Background

Prejudice, as understood in this work, is a complex phenomenon that represents an attitude or set of attitudes displayed by an individual or individuals that can be understood and measured in different ways and that can be affected by factors that are internal (e.g., individual bias) and external (e.g., context) to the subjects who show it. It is possible to discuss aspects of the phenomenon of prejudice at various levels of description. In an attempt to consider the phenomenon of prejudice at the level of its possible relationship to cerebral event related potentials, we developed an experiment in which several measures in addition to EEG are taken in subjects undergoing processes in which social bias is involved, and we then analyzed relationships that arose in those data.

### Explicit vs. implicit assessment of social bias

In social cognition research, it has been regularly assumed that simpler processes are carried out separately from and in advance of more elaborate processes that include the former. Using this interpretation, simpler processes (quick, easy, involuntary and unconscious) would be automatic while more elaborate processes (slower, more difficult, voluntary and conscious) would be more controlled (e.g., [1]). In social psychology (see [2]), this distinction has inspired theories on attitudes, i.e., positive or negative evaluation of stimuli by subjects. For example, it has been proposed that attitudes are based on automatic memory processes involving the association of concepts with evaluative attributes [3]. According to this theory, only if a subject had sufficient cognitive resources (motivation due to high elaboration, time to carry out task in detail, etc.) would important controlled processes of social standards, complex reasoning and eventually correction of perceived biases arising from automatic processes be activated [4,5]. Other distinctions, such as impulsive versus reflexive processes [6] and implicit versus explicit attitudes [3], belong to this same family of dual models (see [7]).

Based on these distinctions, it has been stated in contemporary prejudice research that automatic associative processes should play an important role in biased behavior toward members of an outgroup. Therefore, in such research, it is relevant to consider implicit measurement techniques, i.e., those that reflect introspectively unidentified or inappropriately identified traces of past experience [3]. Explicit answers to questions that assess social bias with respect to an outgroup would be affected by conscious verbal processes tending to correct, or even hide, automatically generated biases that depend on social desirability or personal ideologies. Besides the Implicit Association Test (IAT) [8] that has been used as a measurement tool in the present study, other implicit attitude measures have also been used in social psychology [9-11]. Generally speaking, the distinction between automatic and controlled processes in prejudice has generated interest in possible relationships between these two kinds of processes in determining attitudes toward social groups [12]. Research that used both indirect measures (e.g., the IAT) and direct measures (e.g., self-reports) has revealed only moderate to low correlations between implicitly and explicitly measured prejudice [13-16], thus providing evidence for a dissociation of at least two cognitive processes. However, other elaborations point to a different interaction pattern [17,18] and suggest a more dynamic relationship between the two kinds of processes. In this line, it is commonly assumed by researchers that automatic associations determine early processing, whereas more controlled processes are likely to intervene in late processing. With respect to racial differences, studies have been reported on Blacks and Whites [19,20], Asians, [21], Germans and Turks [22], and Chilean indigenous peoples [23-25].

### Previous ERP research

ERP studies have shown that N170 and VPP early potentials are sensitive to face categorization [26-32], and even racial cues [33,34]. The late positive potential (LPP) has been associated with evaluative categorization and stimulus valence [35,36]. Numerous findings suggest that the LPP presents a higher amplitude when it is evoked by emotionally relevant stimuli, such as images that generate pleasure or displeasure [37-39]. Pastor et al [40] found that the LPP does not vary based on relationships between a similar/different valence context for pleasant and unpleasant image targets, but that it does vary in the case of neutral vs. emotional images, with independence of the context. However, other authors suggest that factors related to perceptual processing of emotional stimuli probably affect early ERP components, but not LPP in terms of valence [41]. In the same line, [36] provided empirical evidence that LPP is actually associated more closely with arousal than with the specific valence of an emotional sign. In [42], a differential effect (LPP) was found between 450 and 700 ms, on the midline and the right frontal scalp. This effect was related to the arousal or alarm level caused by the stimulus. However, LPP measured by central-parietal electrodes in the left hemisphere was modulated by semantic cohesion more than by arousal.

### LPP contextual blending and racial effects

In LPP research, context is well known to affect LPP. Nevertheless, only a few studies have considered contextual effects that imply a combination of different stimuli dimensions (e.g., valence and race). We refer to contextual effects simultaneously present in more than one attribute as "contextual blending". The IAT performed in the present study implies the contextual blending of three different properties: the participant's social position relative to each face stimulus (ingroup or outgroup), the racial category of presented faces, and the valence that the task associates with that category. In addition, the effect of the contextual combination of properties (relative position between stimulus and participant, racial category and associated valence) is actually present in the same slide. In brief, when three attributes (relative position, race and valence) have to be judged together in each trial evaluation, we refer to this effect as contextual blending. The complexity of performing a meaningful categorization of attribute combinations has not been attempted in previous frontal LPP studies [43] and the simultaneous presentation of contextual effects is not present in the sequential oddball paradigm from most LPP contextual effects (e.g., [44]). Several previous reports show a centro-parietal distribution of LPP, usually related to a P3b morphology. Those studies show a contextual effect (P300 larger when current stimulus is inconsistent with the context established by preceding stimuli; [33,34,45]); this outcome was affected in most cases by the sequential dynamics present in an oddball paradigm. Nevertheless, LPP presents a wide variety of morphologies and scalp distributions depending on the paradigm. In particular, LPPs related to evaluative processing have been found to exhibit a frontal localization, in contrast to the classic LPP which has a P3b-like distribution over parietal bilateral sites. This component, different from the classical LPP elicited by oddball-like paradigms, has been observed when participants are required to make judgments on goodness vs. badness (evaluative) or abstract vs. concrete (non-evaluative) properties of socially relevant concepts (i.e., "murder", "welfare"; [43]). In these studies, frontal LPP varied as a function of both stimulus valence and laterality: the amplitude of the right-sided frontal LPP was greater for negative stimuli than positive. Conversely, the amplitude of the left-sided frontal LPP was greater for positive stimuli than for negative. Moreover, the LPP amplitude was larger when participants were making evaluative judgments than when they were making non-evaluative judgments. This LPP not only differs in scalp lateralization compared to the centro-parietal LPP, but it also differs in the parietal LPP non-modulation based on evaluative vs. non-evaluative judgments because those LPP are larger for negative stimuli in a positive context than for positive stimuli in a negative context whether participants are making evaluative or non-evaluative judgments [46,47]). Furthermore, LPP non-modulation occurred based on positive vs. negative stimuli (since LPP is greater over the right hemisphere than over the left for both positive and negative stimuli presented in an incongruous evaluative context, i.e., [44,45], and even using valenced stimuli presented in a random sequence i.e., [38]).

Another important issue regarding ERPs and social bias research relates to whether race-related ERP differences are caused by the physical properties of facial stimuli (i.e., skin tone, hair type, facial features) or by in-/outgroup relative position. Recent research by Dickter & Bartholow [48] using a task-irrelevant racial dimension shows an ingroup attention bias in ERPs facilitating target categorization that is independent of the race-related physical properties of stimuli, suggesting a potential functional role for differentiation of ingroup and outgroup targets. As in the study conducted by Dickter & Bartholow [48], we included in the present study participants and stimuli corresponding to both minority (indigenous) and majority (non-indigenous) social groups, but in a race-relevant task. If the in-/outgroup effects are inversely similar in both groups of participants, those effects should not be explained in terms of physical properties of facial stimuli. Since our paradigm implies an evaluative task with positive and negative valence association, the expected result is a frontal distribution of LPPs similar to that reported by Cunningham et al. [43]. At the same time, we predicted that LPP valence lateralization (positive to the left, negative to the right) would be modulated not only by positive/negative associations but also by combinations of valence, face stimuli and relative position of the participant. If a contextual blending of attributes is present in this task, the compatible and incompatible task (being positively and negatively evaluated differentially from each group) should produce an LPP lateralization in agreement with the valence effects previously reported. Specifically, in indigenous participants, the compatible task that induces a bias against a minority (ingroup-negative association and outgroup-positive association) implies an overall negative evaluation that should increase the right LPPs compared with the incompatible task, which implies an overall positive evaluation. This effect should be inverted in the left LPP since previous reports suggest that positive evaluation implies a higher amplitude compared with the negative evaluation in this region. The compatible/incompatible effect should at the same time show an reversal in non-indigenous participants (because of their expected ingroup favoritism). The lateralized LPP effect should be enhanced when a group shows a stronger behavioral ingroup bias (measured with IAT and explicit questionnaire).

### A multileveled approach to social bias research

The rich landscape presented by prejudice research, which is based on interacting implicit and explicit attitudes of subjects toward different social groups and which is susceptible to measurement with different instruments, is a motivation for studying this phenomenon with a multileveled approach. This is achieved by integrating results from different measures taken simultaneously from the same situation of prejudice. Additionally, with such a goal in mind, it is desirable not to forget the role that context may play in the process. For instance, in face perception, both perceptual and higher-order levels coexist: in a few milliseconds, low level features allow a distinction between the ingroup and outgroup. It is likely that these processes are affected by the context, for instance, by contextual association of positive or negative valence with stimuli; this should at least not be immediately ruled out.

With these concerns in mind, the present study deals with implicit and explicit biases involving Chilean indigenous people, specifically the Mapuche ethnic group. The Mapuche are Chile's largest indigenous group and are at the same time one of the most deprived social groups due to their historical struggle against the Spanish crown and, afterwards, the Chilean Republic (see [49,50]). The Mapuche face negative beliefs about themselves, which pervade Chilean society. Non-indigenous Chilean stereotypes depict them as violent, rude, lazy, and unintelligent [51,52]. Two previous studies on implicit and explicit attitudes including Mapuche participants have been reported [23,24] and show that both types of measures detect bias. In a previous work (Ibáñez A, Hurtado E, González R, Haye A, Manes F: Early neural markers of Implicit Attitudes: N170 modulated by intergroup and evaluative contexts in IAT, Submitted), we examined the early ERP processing of the present data. Indigenous and non-indigenous participants performed an implicit association test (IAT) displaying faces (ingroup and outgroup) and words (positive and negative valence). The N170 ERP component was found to be modulated by the structural features of stimuli (faces and words), the associated valence (positive/negative), and social in-/outgroup association. With respect to N170, association with positive valence generated higher amplitude (*m *= -5.30, *sd *= 0.67) than did association with negative valence (*m *= -4.11, *sd *= 0.47). In the right N170 region, faces generated higher amplitude responses for positive valence stimuli when associated with outgroup categorization (*m *= 8.46, *sd *= 0.50), compared with ingroup/negative association (*m *= -6.61, *sd *= 0.63). Nevertheless, non-indigenous participants did not evidence a main effect of social category association. Therefore, N170 effects not only revealed modulation of valence and race association occurring during early structural processing, but they also suggested contextual integration of both effects (valence and relative social position). In general, indigenous participants reveal more ingroup bias, and therefore various measures show a differential pattern in that direction. This suggests that early on there is a reaction to stimulation that is semantically unfavorable to the group itself. Such reaction is coherent with the difficulty shown by the subjects in tasks that involve working with such stimuli; these results suggest that a crucial aspect of the IAT cognitive task lies in processing stimuli that are contrary to a positive representation of one's own social group. The general implications of our results are consistent with recent research on the contextual malleability of implicit attitudes [53-55] and overall cognition [56-61] (also Ibáñez A, Toro P, Cornejo C, Weisbrod M, Schröder J: High contextual sensitivity of metaphorical expressions and gesture blending: A video ERP design, Submitted). For example, when behavioral measures of ingroup favoritism were stronger (indigenous participants), an increased early contextual modulation of ERPs was observed, suggesting an early influence of contextual effects on IAT measures. These results suggest that high level contextual combinations of social relative position and valence have an early neural modulation and a consequent behavioral effect.

Here we re-analyzed previous ERP data in order to report the late effects (LPP, not reported previously) and to determine their relationships to behavioral results, explicit measures and early responses (N170/VPP). Using this approach, we can relate LPP modulation to several measures of social bias, providing converging information about early and late processing of IAT. Since the N170/VPP early effects were detailed in the previous report, we do not account for the same data here; however, we do use a data mining approach in order to find relationships between that data and data reported here, so as to have a richer landscape and to be able to assess the convergence of multiple measures.

For this study, we expected that (1) implicit social bias measurements (using a race-IAT) would show ingroup favoritism, (2) explicit social bias (assessed by an explicit questionnaire) would either show ingroup favoritism or neutral results (because of a possible conscious correction of automatic bias), and (3) the frontal LPP ERP component expected in an evaluative task like the race IAT would show modulation in terms of what could be understood as "the valence of associations" (whether the task determined association implies a positive or negative evaluation of the ingroup). Previous reports on the relationship between implicit and explicit bias are not conclusive; therefore, we explored relationships between the several variables (including ERP data) without expecting a particular pattern and we found some sound links (see Discussion).

The present study involves several measures of prejudice, including the LPP event related potential as a neural correlate, useful for exploration from a social-neuroscientific perspective. In order to study possible LPP blending between facial race processing and semantic valence (positive or negative words), we recorded ERPs while indigenous and non-indigenous participants matched by age and gender performed an implicit association test with faces (ingroup and outgroup) and words (positive and negative). In addition, explicit attitudes were assessed by means of a questionnaire, and an implicit prejudice measure was extracted from IAT behavioral data. Relationships between different measures were studied by computing correlations. When ERPs were involved, the number of correlation coefficients was high, so a data mining method was used to automatically discover which kind of variables showed a close relationship. This analysis yielded interesting convergences for different measures.

Although previous reports exist of early and late effects of race and valence, to our knowledge there is no report of frontal LPP contextual blending of ingroup/outgroup stimuli, semantic valence and race of participants using an IAT. Our findings may be of use in the context of current research in social cognition concerning both implicit/explicit distinction and ingroup/outgroup relative position.

## Methods

### Participants

Thirty-six Chilean subjects between 18 and 40 years of age participated in the study. Half were of indigenous origin (mean age: 26.2 years, sd: 7.13), and half were non-indigenous (mean age: 25.7 years, sd: 5.7). Of the 18 indigenous participants, 11 were women (61%), and in the non-indigenous group, 7 were women (38%; *t*(34) = 1.329, *p *= 0.1927) [see Additional file 1]. Because of the intrinsic difficulty of reaching indigenous subjects in Chile, there was an unavoidable need to pay them for their participation.

Budget restrictions disallowed also paying subjects in the non-indigenous group. Limitations that arise from this restriction are considered in the discussion section. Each subject who participated in the study read and signed an informed consent document in which the experimental procedure was described. Subjects were absolutely free to decide whether or not to sign the document (and therefore participate), and only mental and physically competent people were considered, not only because of methodological concerns but also to make sure that they were free to decide on their participation. It was stated very clearly that each participant could leave the experiment at any moment that he or she decided to do so. In the case of subjects who were paid for their participation, it was made clear that they would be paid even if they left the experiment, and this included the possibility of leaving before it started. Personnel from our lab constantly monitored subjects, especially during EEG recordings, in case any need arose. This experiment was approved by the Ethical Committee of the Cognitive Neuroscience Laboratory at Universidad Diego Portales. All procedures were performed according to the Declaration of Helsinki [62].

### Procedure

**Figure 1:**
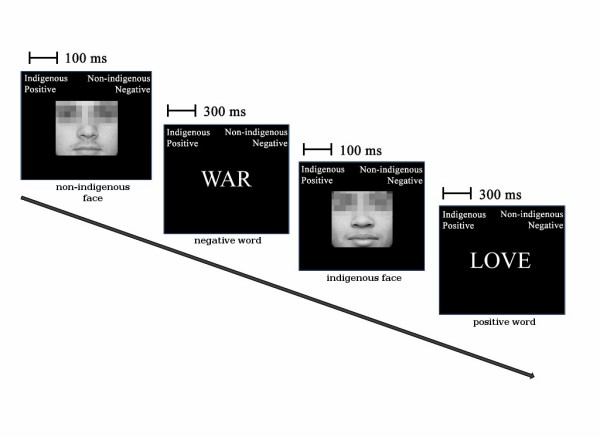
**IAT sequence schematic representation**. Face and word trials are presented for a short time, strictly interleaved. Both indigenous and non-indigenous faces, along with words of positive and negative valence, are present in the stimuli set and are presented in a randomized sequence. The subject is required to classify each stimulus to the left or to the right according to labels displayed on top of the screen. Reaction times and EEG signals are recorded in each session. Pictures are used with consent of shown subjects. Eyes are shown pixelized for anonymity, but were shown undistorted during the experiment.

Initially, participants were informed that the study assessed recognition processes and opinions regarding people and words. For the IAT, after being given instructions, the participants sat in front of a computer with the electrodes placed on their heads and responded to the stimuli displayed on the computer screens. Responses were given by pressing with each forefinger either of two keys on a response pad (see Figure 1). After the IAT was completed, participants were asked to write the answers to the attribute evaluation questionnaire. Once the experiment was completed, participants were thanked and the research goal was thoroughly explained.

### IAT

The test involved the presentation of stimuli taken from a set of indigenous (*N *= 20) and non-indigenous (*N *= 20) faces, along with pleasant and unpleasant words. Ethnical content of pictures and valences of words were validated in a previous study [see Additional file 1]. In the case of pictures, means for red, green, and blue components were measured for each picture. An ANOVA model was fitted to the data, grouping errors by color component (red, green, and blue) but not by ethnicity of the depicted faces, so as to assess differences that arose due to skin tone that was a result of ethnicity but not differences that were a result of color component (such differences will unavoidably be present since images were not in grayscale, i.e., not all three color components have the same intensity). The interaction between group and color components did not show heterogeneous skin tones (*F *(2, 114) = 1.416, *p *= 0.247). Each block of the test included a brief explanation of how each category was assigned to each response key. Subsequently, trials were presented one by one, until the specific number for each block was displayed. The practice blocks involved approximately 28 stimuli, and the test blocks involved 100 stimuli. Faces were displayed for 100 ms and words for 300 ms. Incorrect responses were indicated with an "X" in the center of the screen after the response. The stimuli were centered horizontally and vertically on the screen. For each block, the categories that required a response were displayed on the top left and right-hand corners. The category names were "Indigenous", "Non-indigenous", "Pleasant" and "Unpleasant". A general IAT design was applied [8], with minor modifications. The test was split up into several blocks. Some blocks had indigenous/unpleasant labels at one side of the screen and non-indigenous/pleasant categories at the other. Because of the implicit association "compatible" with prejudice against the indigenous, these are called compatible blocks. Blocks having the opposite association are designated incompatible. The two possible assignments of hands, and the two possible associations (compatible and incompatible) generated four main blocks, two compatible and two incompatible. Each main block was preceded by three practice blocks: one for training face categorization, one for words, and one combining both in a task identical to the corresponding main block but shorter in length. Details can be found in the supplementary data [see Additional file 1].

### Measures

#### Behavioral Measures

##### IAT

An implicit racial bias rate was calculated for each subject based on reaction times obtained from the IAT; this numeric value provides an indicator of the tendency observed in the reaction time difference between compatible and incompatible tasks with racial bias toward indigenous minority. Greenwald, Nosek, & Banaji [63] have proposed a method to calculate this rate that involves eliminating extreme reaction times and includes special management of wrong responses and standardization of resulting reaction times based on the standard of each subject. The method eliminates from the analysis reaction times over 10000 ms and recalculates wrong answers by adding 600 ms to their real values. A subject's rate is obtained by measuring the reaction time difference between compatible and incompatible blocks, standardized according to the standard deviation. Hence, a measurement procedure to evaluate racial bias was achieved using a scale that enables comparison between different subjects and, therefore, between different subject groups, even if there were systematic differences in the reactions of different groups. The step by step procedure that was carried out has been detailed in the supplementary data [see Additional file 1]. A previous algorithm for IAT score computing [8] was used at an early stage of the study. Since computing scores with the newer algorithm [63] did not change any of the conclusions in this study, it was decided to use the latter to achieve comparability with new research. The result of this procedure is a number with an expected value close to zero for subjects who did not show racial bias in the test. Positive values correspond to the detection of bias in favor of the indigenous group, while negative values indicate bias in favor of the non-indigenous group. By the method in which it is calculated, the result is similar to Cohen's d coefficient; hence, it can be interpreted as an effect size measure. In order to enhance the measurement and also to simultaneously record reaction times to faces and words as in the procedure proposed by [63], this study included two additional measures that were obtained separately taking into account face and word trials. Therefore, it was possible to explore in more detail the connection between the IAT behavioral information and the descriptors resulting from the extraction of ERPs, which have different waveforms for words and faces. Separate analyses were performed based on stimulus type (face or word), experimental group (indigenous or non-indigenous) and task (compatible or incompatible with prejudice toward minority), resulting in 8 categories. Finally, in order to obtain accuracy for both groups in this IAT, correct responses were divided by the total number of responses yielding an accuracy score between 0 and 1. Accuracy is calculated based on compatible and incompatible blocks and on the separate responses to faces and words.

#### Explicit Questionnaire

The procedure to measure explicit intergroup attitudes involved an attribute assessment task developed by Brown et al [64], also used in our indigenous participant samples [24,25]. This involved various items that related to "feelings toward minority and majority", "attributes of minority and majority", "culture", "emotions toward majority and minority", "perception of the economic status of the minority", "perception of discrimination toward minority", "similarity between minority and majority", "perception of living conditions of minority", and "nationalism". These items included questions about positive and negative attributes relevant to such categories, applicable to both indigenous and non-indigenous social groups. Participants were required to judge each item based on their beliefs and evaluate the appropriateness of the attributes using a scale from 1 (very little) to 7 (very much). The explicit questionnaire included questions referring to the indigenous group (minority) and to the non-indigenous group (majority). For both types, there were items that required attributions of positive and negative valence. This scheme produced four question categories; the averages for the corresponding responses were four measures obtained in the test. In order to ensure the consistency of each measure, an internal consistency analysis was carried out by calculating Cronbach's alpha coefficient with respect to the four item categories. The analysis was carried out separately for indigenous and non-indigenous subjects. In each category, if the alpha coefficient was lower than 0.7, a component was removed. The removed component was chosen so that its elimination would improve the coefficient as much as possible. In each case, the procedure was repeated until the alpha value was greater than or equal to 0.7. For the indigenous group, answers to questions about negative attributes showed more internal consistency (Cronbach's alpha 0.73 targeting the non-indigenous group and 0.84 when assessing the outgroup). In the non-indigenous group, the only block that had an alpha higher than 0.7 attributed to themselves negative characteristics (Cronbach's alpha 0.77). For the remaining blocks, items were eliminated until Cronbach's alpha was equal to or higher than 0.7. Resulting internal consistency rates can be found in the supplementary data [see Additional file 1].

### Electrophysiological Recordings

**Figure 2:**
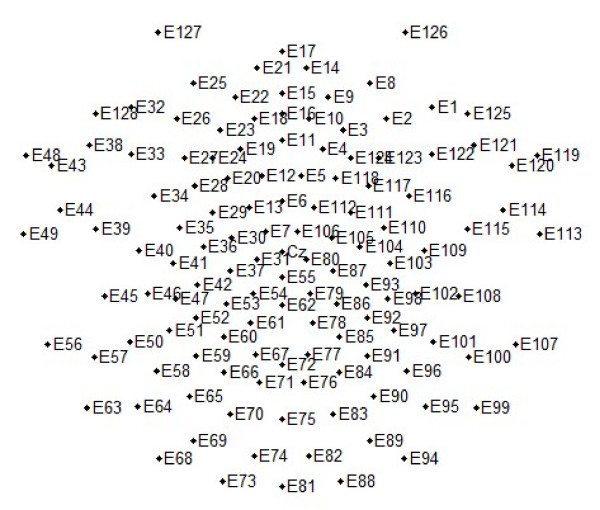
**Channel locations for EGI HCGSN128 electrode net**. Figure shows a schematic representation of EEG electrode localizations over the scalp for the EGI HCGSN128 net used within this study.

EEG signals were sampled at 500 Hz from 129 electrodes. Data outside the frequency band between 0.1 Hz and 100 Hz were filtered out during the recording. Later, a band-pass digital filter between 0.5 and 30 Hz was applied to remove unwanted frequency components. During recording, the reference was set by default to vertex, but afterwards, it was re-referenced off-line to average electrodes. Two bipolar derivations were designed to monitor vertical and horizontal ocular movements (EOG). Continuous EEG data were segmented from 200 ms prior to 800 ms following the stimulus. All segments with eye movement contamination were removed from further analysis, using automatic (Gratton, Coles, and Donchin method for removing eye-blink artifacts) and visual procedures. Artifact-free segments were averaged to obtain the ERPs. The analysis was performed separately based on the following factors: (1) stimulus type (face or word), social category association (indigenous or non-indigenous; corresponds to the social category label at the screen side of the right answer) and valence association (positive or negative; corresponds to the valence label at the side of the right answer), resulting in 8 categories: Indigenous picture in an Indigenous/Pleasant context; Non-indigenous picture in a Non-indigenous/Pleasant context; Indigenous picture in an Indigenous/Unpleasant context; Non-indigenous picture in a Non-indigenous/Unpleasant context; Positive word in an Indigenous/Pleasant context; Positive word in a Non-indigenous/Pleasant context; Negative word in an Indigenous/Unpleasant context; Negative word in a Non-indigenous/Unpleasant context. ERP waveforms were averaged separately for each experimental condition. The EEGLAB Matlab toolbox [65] and T-BESP software  were used for EEG off-line processing and analysis. Regions of interest (ROI) were used to analyze the scalp topography of the ERP component; this is recommended for dense arrays since it improves statistical power [66]. ROIs were chosen by visual inspection of each component. Each LPP ROI was grouped from 7 adjacent electrodes (see figure 2): LA (Left Anterior ROI: 25 26 27 32 33 38 128); RA (Right Anterior ROI; 1 2 8 121 122 123 125); CZ (Central midline ROI: 5 6 11 12 13 112 129); LP (Left Posterior ROI: 58 63 64 65 68 69 73) and RP (Right Posterior ROI: 88 89 90 94 95 96 99). Additionally, N170/VPP early potentials were extracted (reported in Ibáñez A, Hurtado E, González R, Haye A, Manes F: Early neural markers of Implicit Attitudes: N170 modulated by intergroup and evaluative contexts in IAT, Submitted). Although the early ERP data are not analyzed here on their own, they are correlated with explicit questionnaire and IAT scores and are analyzed using the data mining scheme described in the following section. For this purpose, the same ROIs used in the previous report were considered: VPP (129 7 106 13 6 112 12), N170 LEFT: (57 58 65 63 64 69 68), and N170 RIGHT (100 96 90 95 89 99 94). Although signal plots show the ERP grand averages from each data cell, statistical contrasts were performed separately considering data for each participant. For ERP analysis, the 350–750 ms time window was visually selected for mean amplitude analysis.

### Data mining of correlations between ERPs and behavioral measures

The IAT procedure captured measures of two types: scores based on reaction times and ERPs. In turn, a third source of information was an explicit bias index calculated from the explicit questionnaire. The relationships between variables from ERPs and each of the other two sources of information were abundant and therefore difficult to interpret once evaluated. Correlations were calculated exhaustively, recording which variable pairs exhibited linear correlations with absolute values larger than 0.3. This threshold was decided after looking at histograms for the correlation coefficients and choosing a low threshold, so as to include weak relationships in the analysis. The analysis was restricted to pairs in which one and only one of the two correlated variables was derived from ERP information. The procedure was performed separately for IAT scores and for questionnaire results. In order to avoid the bias present in the visual selection of a time window, for the data mining analysis only ERP information was obtained from peak amplitude and latency instead of averaged window values. This is a commonly used approach when computing correlations with ERP data [67,68]. For the extraction of peak and latency values from ERP data, the peak detection transform of Brain Vision software was used. This function searches for local minima and maxima (global maxima and minima interval) in a segment of each trial in each electrode; these are detected and marked by a semiautomatic module of Brain Vision software. Extracted information was visually checked looking at waveforms with a cursor indicating the position at which the algorithm detected the peak. In order to detect patterns, pairs of correlated variables were arranged in a table with one row for each pair, with some columns indicating which ERP measurement was being correlated and the rest identifying the other variable in the correlation. In order to avoid personal biases when exploring these data, the Apriori algorithm [69] was used to automatically extract association rules, that is, relationships that tend to appear in several rows (or several pairs of correlated variables in this case). Each association rule is expressed as a causal relationship where values for some columns determine either fully or partially values for other columns. Although this data mining method was inspired by shopping basket analysis, there have been applications to analysis of web application activities [70] and also to biochemistry and particularly to genetic analysis [71,72]. The Apriori algorithm is often applied to databases containing thousands of observations. However, in this study, the number of rows in the tables that are analyzed is in the hundreds. Despite the fact that the number of input observations in our study is lower than usual for this method, each observation is very reliable because it is based on a correlation that in turn aggregates several observations of ERP values and other variables. Because of this, it is unlikely that the patterns extracted with this procedure are due to mere chance or that they are unrelated to the phenomenon being studied. Furthermore, in order to assess its stability, the procedure was applied separately for indigenous and non-indigenous groups of subjects. Though correlations varied, tables recording only rows with correlations larger in magnitude than 0.3 were exactly the same for both groups in ERP-IAT. Since the two groups of subjects are completely separate, this result is extremely unlikely to be observed by chance, and it speaks to the generality of the procedure. These factors having been considered, the Apriori algorithm is deterministic and provides a good aid for exploring, though not confirming, systematic patterns that appear in the abundant but consistent correlations that were computed.

## Results and Discussion

The following subsections provide descriptive statistics for the measures obtained with each of the previously described instuments. Also, it is stated whether differences of means are statistically significant in each case. Further interpretation and integration can be found in the Discussion section.

### Behavioral results

#### Explicit questionnaire

**Figure 3:**
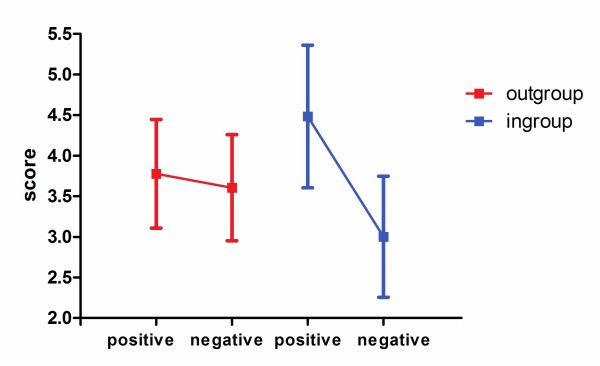
**Explicit questions scores by relative social position and valence**. The vertical axis shows explicit questions score values. The horizontal axis displays valence: positive and negative for both, outgroup and ingroup relationship of the subject with the question target. Interaction is relevant (*F *(1, 68) = 10.2805, *p *= 0.0017). Post-hoc comparison yields significant positive vs. negative differences for outgroup position (*p *= 0.7567) but not for ingroup (*p *< 0.0001).

**Figure 4:**
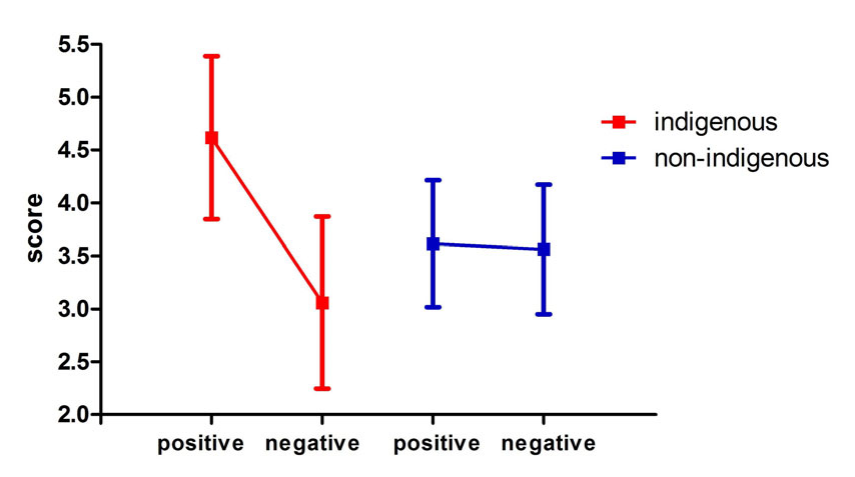
**Explicit questions scores by participant group and valence**. The vertical axis shows explicit questions score values. The horizontal axis displays valence: positive and negative for both, indigenous and non-indigenous participants. There is an interaction effect (*F *(1, 68) = 52.8548, *p *< 0.0001). Post-hoc comparison yields a positive vs. negative difference for indigenous participants (*p *< 0.0001) but not for non-indigenous (*p *= 0.9884).

Questionnaire scores were analized by subject group, attribute valence, and target social category. Questionnaire scores given by indigenous subjects (*m *= 3.8379, *sd *= 1.1107) were significantly higher than those given by non-indigenous subjects (*m *= 3.5883, *sd *= 0.6016) (*F *(1, 34) = 5.6867, *p *= 0.0184, eff-size = 0.3975). As expected, regardless of the group, the participants tended to give higher scores to questions about positive attributes (*m *= 4.1297, *sd *= 0.8532) compared to questions about negative attributes (*m *= 3.3033, *sd *= 0.7596) (*F *(1, 68) = 14.5065, *p *= 0.0002, eff-size = 0.6348). It was noted that the scores were subject to an interaction effect (see Figure 3) for valence and the target social category (*F *(1, 68) = 10.2805, *p *= 0.0017, eff-size = 0.5344). In general, when the questions referred to the outgroup, there were no significant differences based on valence (positive: *m *= 3.7763, *sd *= 0.6692; negative: *m *= 3.6055, *sd *= 0.6533; positive-negative: *p *= 0.7567). On the contrary, there were differences when the questions were about the ingroup (positive: *m *= 4.4830, *sd *= 0.8783; negative: *m *= 3.0011, *sd *= 0.7452; positive-negative: *p *< 0.0001). There was also an interaction efect (see Figure 4) for participant group and valence (*F *(1, 68) = 52.8548, *p *< 0.0001, eff-size ≈ 1). Indigenous subjects gave higher scores when asked about positive-valence adjectives (*m *= 4.6174, *sd *= 0.7708) than negative-valence adjectives (*m *= 3.0584, *sd *= 0.8114) (indigenous.positive-indigenous.negative: *p *< 0.0001). This trend was not detected in non-indigenous subjects (positive: *m *= 3.6149, *sd *= 0.5993; negative: *m *= 3.5618, *sd *= 0.6111; nonindigenous.positive-nonindigenous.negative: *p *= 0.9884). It is worth noting that despite the statistically significant variations, all the score means presented here were around 4.0, which is expected since possible answers were from 1 to 7. Also, standard deviations were lower than 1.0. It is therefore unlikely that floor or ceiling effects are present in this data.

#### IAT

##### Accuracy

An ANOVA model was fitted to correctness of responses with stimuli type (face or word) and task (compatible or incompatible) as within-subject factors and group as a between-subject factor. The non-indigenous group had higher accuracy (*m *= 0.840, *sd *= 0.017) than the indigenous group (*m *= 0.727,*sd *= 0.017) when answering the IAT (*F *(1, 34) = 22.6087, *p *< 0.0001, eff-size = 0.7925). An interaction effect was found between participant group and task type (*F *(1, 68) = 4.8515, *p *= 0.0312, eff-size = 0.3671). Post hoc comparisons revealed that when non-indigenous subjects responded to the compatible task, they had higher accuracy (*m *= 0.842,*sd *= 0.016) than in the incompatible task (*m *= 0.837, *sd *= 0.017; nonindigenous.compatible-nonindigenous.incompatible: *p *< 0.0001). On the contrary, the indigenous group obtained lower accuracy (*m *= 0.674, *sd *= 0, 0165) in response to compatible tasks than to incompatible tasks (*m *= 0.779, *sd *= 0.0171; indigenous.compatible-indigenous.incompatible: *p *< 0.0001).

##### IAT scores

**Figure 5:**
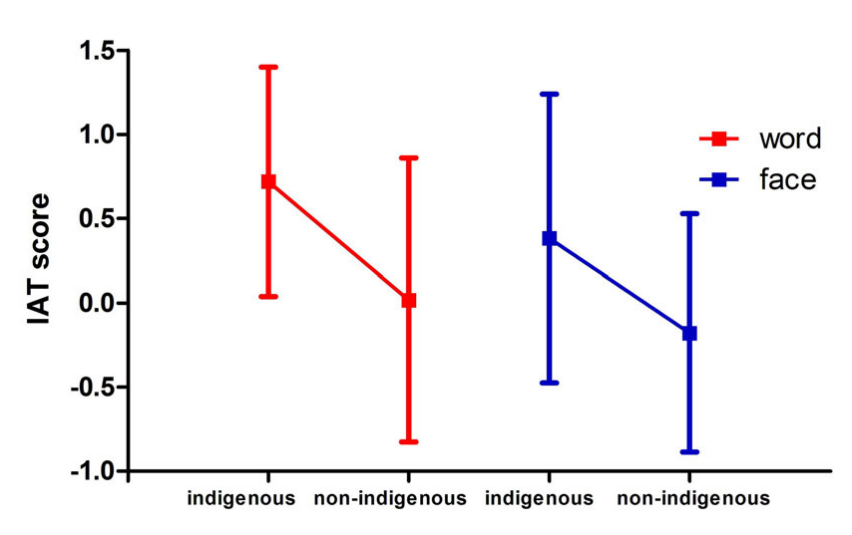
**IAT scores with data split by stimulus type**. The vertical axis shows IAT scores. The horizontal axis displays participant groups: indigenous and non-indigenous participants in both word and face stimuli categories. Single sample t-tests were performed to test for difference against zero. Only word scores for indigenous participants yielded a significant offset from zero (*p *= 0.0003).

As for the scores obtained in the IAT (see Figure 5), the indigenous group obtained an average score of 0.4687 (*sd *= 0.5793). This value was significantly higher than zero (*t *= 3.4331, *df *= 17, *p *= 0.0032), which means that this group took more time to respond to the compatible than the incompatible task. The opposite was observed in the non-indigenous group, which obtained an average of -0.0752 (*sd *= 0.7331), although in this case the value was not significantly different from zero (*t *= -0.4352, *df *= 17, *p *= 0.6689). These results show that the applied test detected racial bias in the indigenous group and not in the non-indigenous group. With respect to word stimuli, the indigenous group obtained an average score of 0.7196 (*sd *= 0.6805), significantly different from zero (*t *= 4.4861, *df *= 17, *p *= 0.0003), while the non-indigenous group obtained an average of 0.0167 (*sd *= 0.8432), which is not significantly different from zero (*t *= 0.084, *df *= 17, *p *= 0.934). In terms of face stimuli, the indigenous group obtained an average score of 0.3823 (*sd *= 0.8573), whereas the non-indigenous group obtained an average of -0.1789 (*sd *= 0.7074). These face scores were not significantly different from zero for the indigenous group (*t *= 1.8919, *df *= 17, *p *= 0.0757) or for the non-indigenous group (*t *= -1.073, *df *= 17, *p *= 0.2982). When Holm adjustment for the six resulting p-values, only the indigenous group's IAT score for words (*p *= 0.0020) and the general score for the same group (*p *= 0.0159) were significantly different from zero. Also, Holm adjusted p-values where obtained for t-tests comparing indigenous vs. non-indigenous scores computed for words (*t *= 2.7609, *df *= 32.537, *p *= 0.0282), faces (*t *= 2.141, *df *= 32.822, *p *= 0.0398), and both (*t *= 2.4726, *df *= 32.289, *p *= 0.0377). All three contrasts yielded significant differences between indigenous and non-indigenous scores, with word IAT scores having the highest significance.

##### Correlations between different behavioral measures

Tables 1 and 2 refer to the linear correlation between each index taken for the IAT and the response averages for each category in the explicit questionnaire. The IAT scores appear inverted (multiplied by -1) for the non-indigenous group. In the indigenous group, the IAT bias scores only showed correlation relevant to questions about positive attributes for the same group (*r *= 0.4886), whereas for the non-indigenous group, all the correlations were of significant magnitude. For the indigenous group (ingroup), the correlation was positive for positive attributes (*r *= 0.4886) and negative for negative attributes (*r *= 0.1833), which showed a consistency between IAT bias and explicit answers. In the non-indigenous group, there was also an IAT bias consistency with the questionnaire for the questions that referred to indigenous groups (outgroup) (*r *= 0.2855 for positive attributes and *r *= 0.4184 for negative attributes). In general, correlations of IAT scores were stronger for explicit questions that assessed the ingroup than for questions referring to the outgroup.

**Table 1:** Linear correlations between IAT scores and explicit question scores for indigenous participants

	Indigenous target category		Non indigenous target category	
	Positive	Negative	Positive	Negative
General IAT	0.4886	-0.1833	0.1928	-0.1045
Word IAT	0.3253	-0.2042	0.0069	-0.1793
Face IAT	0.4904	-0.1067	0.2314	0.0169

For each subject, answers were averaged in the four categories shown as table columns. Also, word, face and general (both) IAT scores were obtained for each subject. The table shows linear correlations for IAT-explicit pairs of variables considering only indigenous participants.

**Table 2:** Negative of linear correlations between IAT scores and explicit questions scores for non indigenous participants

	Indigenous target category		Non indigenous target category	
	Positive	Negative	Positive	Negative
General IAT	0.2855	-0.4184	0.3532	0.4734
Word IAT	0.2728	-0.3467	0.3722	0.4624
Face IAT	0.2910	-0.4590	0.3182	0.4427

For each subject, answers were averaged in the four categories shown as table columns. Also, word, face and general (both) IAT scores were obtained for each subject. The table shows linear correlations for IAT-explicit pairs of variables considering only non-indigenous participants. Correlations have been multiplied by -1 for easier comparison with table 2, since original IAT scores are referenced not to outgroup prejudice but to indigenous group prejudice.

### ERPs

LPP amplitudes were subject to an effect of stimuli type. Words produced amplitudes closer to zero (*m *= -0.28, *sd *= 0.95) than faces (*m *= -0.59, *sd *= 0.34; *F *(1, 34) = 12.688, *p *= 0.0012, eff-size = 0.5937). ROI also had a main effect with *F *(4, 136) = 31.5270, *p *< 0.0001, eff-size = 0.9358 (LF: *m *= 0.1, *sd *= 0.34; RF: *m *= 0.3, *sd *= 0.42; Cz: *m *= 0.89, *sd *= 0.46; LP: *m *= -1.38, *sd *= 0.39; RP: *m *= -2.04, *sd *= 0.54), and an interaction effect occurred between ROI and group (*F *(4, 136) = 7.5862, *p *< 0.0001, eff-size = 0.4591; higher amplitudes in CZ, LP and RP in the indigenous group). Interestingly, there were no main effects or interactions involving the social category association (ingroup-outgroup). There was an interesting interaction effect between valence and ROI (*F *(4, 136) = 3.0970, *p *= 0.0180, eff-size = 0.2933). Post Hoc comparisons (LSD test, Bonferroni Corrected, *M S *= 5.1285, *df *= 136) revealed significant differences for ROI in LF and RP (left-frontal and occipital-right bipolar voltage pattern): higher amplitudes for positive valence in the left frontal region (*m *= 0.64,*sd *= 0.34) compared to negative valence (*m *= -0.22, *sd *= 0.45; *p *= 0.0053) and a tendency for the same pattern but reversed in the right occipital region of the stimuli associated with positive valence (*m *= -1.89, *sd *= 0.34) versus negative valence (*m *= -2.34, *sd *= 0.54; *p *= 0.0562). ANOVA was again fitted, reworking the social category factor (using indigenous/non-indigenous as levels instead of ingroup/outgroup); no main effect for this factor (*F *(1, 34) = 2.9432, *p *= 0.0959, eff-size = 0.2859) or interaction was observed. Given that, in the previous ANOVA, main social category effects or interactions were also not observed, the new results are similar. Since in the LPP analysis there was no race effect observed, and given that this component has been directly related to expressing racial bias [73], it was analyzed based on COMPATIBLE categories with prejudice toward indigenous minority (faces and words associated with Non-indigenous-Positive and Indigenous-Negative) and INCOMPATIBLE with prejudice toward indigenous minority (faces and words associated with Non-indigenous-Negative and Indigenous-Positive).

**Figure 6:**
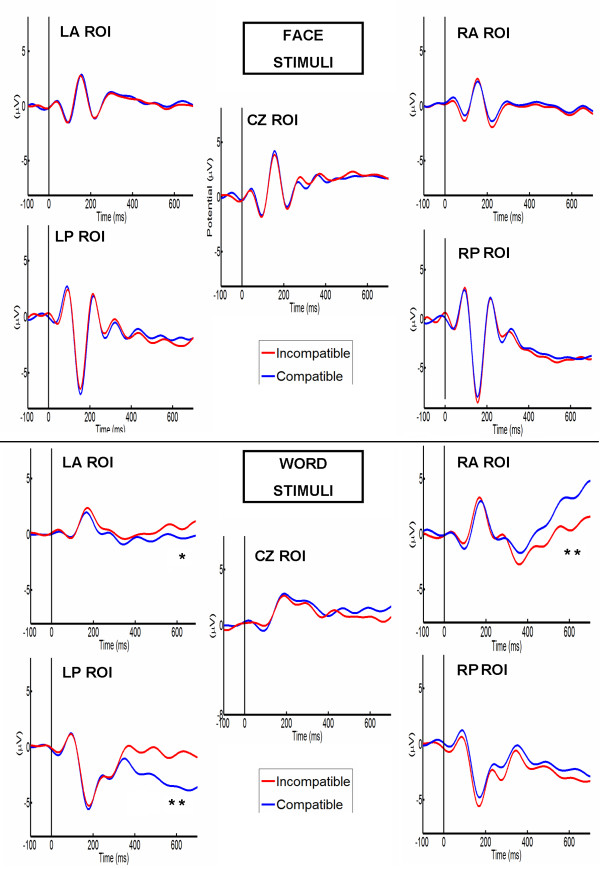
**Indigenous participants LPP ERPs**. LPP selected ROIs for face and word stimuli compatible (Blue) vs. incompatible (Red) with prejudice against the indigenous minority. Top: ERPs of Faces. Bottom: ERPs of Words. In agreement with the behavioral results, we found a LPP modulation in the right frontal areas (and a bipolar voltage pattern on left posterior ROI) related to compatible blocks with prejudice to the indigenous only in the Word Condition in Indigenous participants. Abbreviations: LA ROI (Left anterior region of interest); RA ROI (Right anterior region of interest); CZ ROI (Vertex region of interest); LP ROI (Left posterior region of interest); RP ROI (Right posterior region of interest). (* = *p *< 0.05). (** = *p *< 0.01).

**Figure 7:**
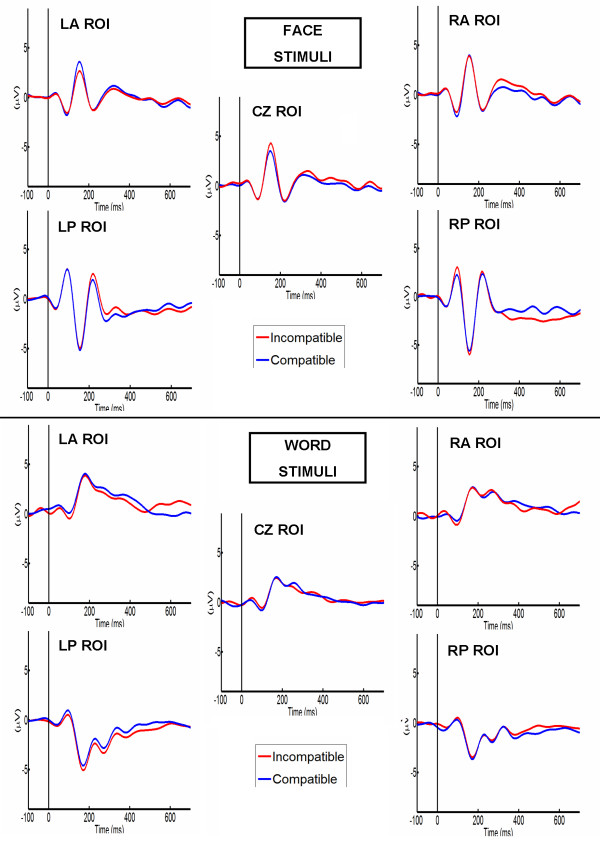
**Non-Indigenous participants LPP ERPs**. LPP selected ROIs for face and word stimuli compatible (Blue) vs. incompatible (Red) with prejudice against the indigenous minority. Top: ERPs of Faces. Bottom: ERPs of Words. Abbreviations: LA ROI (Left anterior region of interest); RA ROI (Right anterior region of interest); CZ ROI (Vertex region of interest); LP ROI (Left posterior region of interest); RP ROI (Right posterior region of interest).

Table 3 shows the most important ANOVA results. The four-factor interaction reported in Table 3 shows that compatible and incompatible categories only produced significant differences for word stimuli in the indigenous participant group (Figure 6; compare to Figure 7). Post hoc comparisons (LSD test, Bonferroni Corrected, *M S *= 2.7612, *df *= 136) showed that, only in this condition, words associated with the compatible block had significantly higher amplitude in the right anterior ROI than incompatible one (Compatible: *m *= 3.01, *sd *= 0.43, incompatible: *m *= -0.94, *sd *= 0.54; *p *< 0.0001). An effect was observed (pattern reversal of previous ROI) in the left occipital ROI (compatible: *m *= -0.19, *sd *= 0.67; incompatible: *m *= -2.63; *p *< 0.0001). In addition, a marginal effect was observed in the left frontal region (compatible: *m *= 0.12, *sd *= 0.34, incompatible: *m *= 1.1, *sd *= 0.41; *p *= 0.0495). The remaining pairwise comparisons from the ROI analysis did not reveal significant differences based on compatibility with prejudice toward minority.

**Table 3:** ANOVA table for LPP peak values

	Sum Sq.	DF	F	p
Stimulus Type	30.9259	1	13.9877	0.0007
ROI	880.8759	4	37.2332	0.0000
ROI * Participants	207.2934	4	8.7619	0.0003
Stimulus Type * ROI	56.3198	4	2.8877	0.0249
Prejudice * ROI	37.9966	4	3.3537	0.0120
Prejudice * ROI * Participants	54.9694	4	4.8518	0.0011
Stimulus Type * Prejudice * ROI	48.7215	4	4.1949	0.0037
Stimulus Type * ROI * Prejudice * Participants	34.6307	4	2.9817	0.0218

Factors are: stimulus type (face or word), ROI (region of interest), participants (indigenous or non-indigenous), and prejudice (compatible or incompatible). Only effects for which p-values are lower than 0.05 are shown.

### Correlations between Behavioral and ERP Measures

Analysis based in the application of the Apriori algorithm was performed separately for IAT-ERP and Questionnaire-ERP relationships. In contrast to other analysis in this text, ERP data were included not only for LPP but also for N170/VPP data (Ibáñez A, Hurtado E, González R, Haye A, Manes F: Early neural markers of Implicit Attitudes: N170 modulated by intergroup and evaluative contexts in IAT, Submitted). In this manner, it is possible to determine whether the relationships found are specific to LPP or general to all the extracted ERP features.

#### IAT-ERPs

For linear association between IAT and the extracted ERP features, association rules were extracted by the Apriori algorithm. The following patterns were found [see Additional file 1]. Four rules show that a remarkable set of correlations between face IAT and ERP occurred in the incompatible task for non-indigenous subjects and in the compatible task for indigenous subjects. In other words, peak values for different ERP waves showed correlations of relevant magnitude (larger than 0.3) with face IAT scores, in IAT trials having ingroup-negative and outgroup-positive associations. Such ERP peaks mainly occurred in response to faces. VPP peak values that correlate well with IAT scores were always elicited in response to words.

#### Explicit Questionnaire-ERPs

For the correlations between questionnaire scores and ERP characteristics, the Apriori algorithm was applied again for the extraction of association rules. The following patterns were found [see Additional file 1]. Correlations between ERP measures and questions about the outgroup (specially of positive valence) occurred with reference to the non-indigenous group. This means that, in the indigenous group, a set of relevant correlations was found between ERP measures and questions about the non-indigenous group. Correlations between face ERP measures and explicit questions were found for questions about the non-indigenous group. The most frequently correlated ERP feature was peak value.

## Discussion

### IAT Behavioral data

The fact that indigenous subjects displayed better accuracy than non-indigenous subjects is less relevant in this study than is the fact that both groups performed better (in terms of accuracy) in the task that was favorable to them. This is to be expected if the IAT is regarded as requiring less effort in the blocks that associate a subject's social group with a positive valence. With respect to IAT scores, the finding of positive scores in indigenous subjects and negative scores in non-indigenous subjects implies that subjects of both groups took less time to answer trials in blocks favorable to their own social group. Nevertheless, this pattern yielded statistical significance only for indigenous subjects, more specifically, only with respect to reaction times in word trials. Compared to other IAT studies (e.g., [74]) our sample size is much smaller, due to the need to record EEG signals for all subjects. The small sample size may explain why only a specific category of IAT scores achieved statistical significance. A meta-analysis [75] has found that ingroup bias would increase with group salience and slightly decrease with status. Since the Mapuche are a low-status minority, this would predict that ingroup bias should be stronger in the indigenous group of our study compared to the non-indigenous group, which is positioned as a higher-status majority. Such ingroup bias could easily explain the larger IAT effect found in the indigenous group. Social Identity Theory [76], a well-established explanation of how prejudice can arise from intergroup relations, emphasizes that members of a group tend to favor the ingroup over the outgroup because they seek positive self-esteem [77]. This is to say that social bias is better explained by a positive evaluation of the ingroup than by devaluation of an outgroup. This is consistent with results from the explicit questionnaire in our study. Regarding the absence of IAT effect in faces, it is a well-known fact that word-IATs yield higher magnitudes of effect than do picture-IATs [78]. An alternative explanation could be that face stimuli remain on the screen for too short a time for discrimination to occur. In fact, short face presentation times are a requirement for the extraction of good ERPs. Nevertheless, it is unlikely that this factor prevented faces from being discriminated. A one-sample t-test shows that mean accuracy for faces (0.7747222) is significantly different from the 0.5 value that would be expected if subjects could not determine the ethnicity of faces (*t *= 13.0404, *df *= 71, *p *< 0.0001). Finally, it is necessary to admit that payment given only to indigenous subjects for their participation may have affected their motivation, possibly contributing to an explanation of why only they showed an IAT effect. However, this is inconsistent with the fact that indigenous subjects had considerably lower accuracy than non-indigenous.

### Explicit Questionnaire

Questions referring to positive attributes received higher scores than negative attributes. More interestingly, an interaction was found between valence and social target category, showing an ingroup favoritism. More specifically, a positive explicit evaluation of the ingroup was found, while the evaluation of the outgroup did not show a statistically significant pattern towards either favoritism or derogation. As for differences between indigenous and non-indigenous subjects, the former gave more valence-guided scores: they gave higher scores to questions about positive attributes, a pattern not so present in non-indigenous subjects.

### Relationship between IAT and Questionnaire

Indigenous subjects only showed relevant correlation of that measure with questions about positive attributes of themselves. Also, more correlations were relevant in non-indigenous subjects. These results clarify several aspects of the study. First, data from indigenous subjects show that larger ingroup bias is related to better ingroup evaluation; this is not the case with worse outgroup evaluation. This result points in the same direction as the explicit questionnaire information alone. Second, non-indigenous subjects showed high consistency between the implicit (IAT) and explicit (questionnaire) measures of prejudice, while indigenous subjects showed lower consistency, evident only in one aspect. This argues against the hypothesis that indigenous subject payment may explain group differences. Even though other measures show no effects in non-indigenous data, the consistency exhibited between two instruments suggests that the absence of effect is not a result of randomness due to lack of motivation, but a consequence of a truly different pattern between indigenous people and our non-indigenous sample. Third, this observation is important not only because it helps rule out a possible limitation of this study but more importantly because it demonstrates that implicitly and explicitly measured prejudice can be associated or dissociated in different social groups (as others have suggested [17,18]). The only correlation that pointed in the opposite direction than expected was found in non-indigenous subjects, where higher implicit ingroup bias correlated positively with negative self- evaluation.

### ERP Analysis

In LPP, there were no integration effects of valence association/social category association as in N170 (Ibáñez A, Hurtado E, González R, Haye A, Manes F: Early neural markers of Implicit Attitudes: N170 modulated by intergroup and evaluative contexts in IAT, Submitted). However, analysis of the compatible categories with prejudice toward the indigenous minority (faces and words associated with Positive-Non-indigenous and Negative-Indigenous) and incompatible with prejudice toward the indigenous minority (faces and words associated with Negative-Non-indigenous and Positive-Indigenous) had an important effect on the group of indigenous participants. In this group, modulation occurred only for words. This modulation was also in keeping with the negative-right frontal pattern reported in semantic valence studies; Cunningham et al. [43] found that LPP was lateralized to the right with concepts later reported on a behavioral level as negative and to the left for concepts that were then rated as positive. In our data, modulation depends on the combination of valence and membership type relevant to compatibility with prejudice toward minority. This corroborates other research showing that LPP amplitude may be more likely to be associated with arousal than with specific valence of an emotional sign [36]. Likewise, it is indirectly related to findings that suggest modulation of the LPP component based on congruence of the stimuli [79] and relationship with measures of prejudice [73]. This result, in particular, places LPP as an electrophysiological correlate of IAT behavioral measures.

Our results demonstrated a stronger right LPP modulation-related evaluative categorization in tasks with respect to valence words implicitly associated with an ingroup devaluation and outgroup positive association. More importantly, participants with stronger ingroup bias (behaviorally measured using IAT responses and explicit questionnaire) showed stronger LPP modulation based on compatible vs. incompatible blocks. No LPP modulation was observed in the Face conditions, which do not imply an evaluative judgment of valence. In contrast to early effects seen in an experiment reported elsewhere (Ibáñez A, Hurtado E, González R, Haye A, Manes F: Early neural markers of Implicit Attitudes: N170 modulated by intergroup and evaluative contexts in IAT, Submitted), we find a comparatively late face-stimuli amplitude reduction. Those results, although somewhat controversial, can be explained in terms of previous reports showing that LPP (frontal [43], posterior [44]) is amplitude enhanced by evaluative judgments. Since words implicate an evaluative judgment of valence and faces do not, we interpreted this difference in terms of an explicit evaluative effect. Our results suggest a frontal, lateralized LPP that is sensitive to judgments of valence and that differs from the posterior LPP and P300. As discussed in the introduction, we expected a frontal distribution of LPP in response to the evaluative process, similar to the LPP reported by Cunningham et al. [43]. Certainly, we find a more frontal LPP, left lateralized for positive stimuli and right lateralized for negative stimuli. More importantly, we find a right lateralized effect of task (compatible larger than incompatible) and a left lateralized trend for the same factor (incompatible larger than compatible) only in the participants who showed more implicit and explicit ingroup bias (indigenous group). Specifically, when participants manifested a strong ingroup bias, the LPP became more lateralized (more amplitudes for positive association to the left and negative to the right) even with more complex stimuli (specific in/outgroup association to positive/negative valence). Since compatible blocks imply a negative judgment of the ingroup for indigenous participants, the LPP increased the amplitude over right frontal areas. Conversely, this effect was inverted in the left frontal area (Incompatible task-a positive association to the ingroup and a negative to the outgroup), suggesting a positive-related LPP processing. Blocking the faces failed to show this effect since the faces do not imply an evaluative judgment of valence.

Our results show that performance of an evaluative task based on valence judgments elicits a frontal and lateralized LPP, confirming previous reports of Cunningham et al. [43] with respect to frontal instead of parietal localization of LPP, valence lateralization (positive to the left; negative to the right), and increment of LPP amplitude related to evaluative judgments of valence compared to non-evaluative judgments of valence. Results of both reports suggest that frontal LPP provides a marker of evaluative processing in which motivationally significant stimuli affect lateral regions of the pre-frontal cortex relatively specialized for processing of positive and negative stimuli (e.g., [43,80]). In addition, our results suggest that the valence lateralization can be elicited even with more complex stimuli (i.e., valence associated with membership) if the participant shows specific bias towards the content of those stimuli.

Numerous studies have investigated the time course of different aspects of evaluative processing from very different perspectives. Several reports suggest that basic evaluative processes are performed fast and automatically (i.e., [81-83]), suggesting that these effects occur prior to awareness attitude information processing (i.e., [3,84,85]). Nevertheless, little is known about the neural processes recruited in such an automatic evaluative process. Recently, electrophysiological studies have shown that basic categorization of race may take place as early as 120 ms (i.e., [33,34]); other studies have shown that ingroup members can be differentiated from outgroup members very early (i.e., [73]). Kubota and Ito [86] recorded ERPs while participants made racial and emotional categorization judgments of Black and White men posing with happy, angry, or neutral expressions. They found that processing of racial and emotional cues occur independently and in parallel, and relatively early in processing. The N200 was modulated by race and simultaneously by emotional expressions. Willadsen-Jensen and Ito [87] studied participants who viewed racially ambiguous faces as well as faces of Whites, Asians, and Blacks while ERPs were recorded. Initial processes (ERPs within 200 ms) showed that racially ambiguous faces were not differentiated from White faces. Around the LPP time window, racially ambiguous faces were differentiated from White faces; however, the racially ambiguous faces were still perceived as more similar to Whites than to Asians or Blacks, suggesting that the degree of ambiguity in racial perception can affect the timing of processing. In a previous report more similar to our study, Banfield et al [88] investigated the electrophysiological components of response inhibition in a Go/NoGo association task (GNAT), using a classic fruit/bugs design that had previously yielded solid behavioral results [11]. Like IAT, the GNAT is a tool designed to measure an individual's implicit attitudes towards certain objects or categories of people. In this task, subjects must inhibit their initial and automatic evaluations in order to complete the task accurately. In studies of the classic Go/No GO effect, these investigators found delayed negativity in incongruent (i.e., insect and good word association) as compared to congruent trials (i.e., insect and bad word association). The present work is the first study demonstrating ERPs modulation based on implicit association between two conceptual categories. In a previous article, we reported the early effects (N170/VPP components) of this experiment (Ibáñez A, Hurtado E, González R, Haye A, Manes F: Early neural markers of Implicit Attitudes: N170 modulated by intergroup and evaluative contexts in IAT, Submitted). In brief, we find in-/outgroup and positive/negative valence discrimination in the N170 component. In both groups of participants (indigenous and non-indigenous), an associative combination of membership and valence modulated the early structural process. These studies support ERP modulation based on evaluative process, general and membership-specific, providing an excellent shortcut between neuroscience and social psychology.

Our results are relevant for ingroup/outgroup research assessed with ERPs. In a previous design, Dickter & Bartholow [48] used an adapted version of the Eriksen flanker task where targets are simultaneously presented with "flanker" stimuli (distractors), to which participants are instructed not to attend. Flankers can elicit either the same response as the target (i.e., compatible trials) or an opposing response (i.e., incompatible trials). Dickter & Bartholow used flanker faces which elicit conflict when their race or gender is incompatible with the target, eliciting a N200 component linked to conflict detection [89]. They found that both target gender and target race, as well as an interaction of target race and participant race (ingroup-outgroup effects), affected the early and P300 components. Context effects studied with oddball-type paradigms typically emerge in the P300 component [33], which is sensitive to trial-by-trial changes in stimulus features. However, in this paradigm (as well as in our design), the context is integrated with the stimuli. As in our report, this study found a reverse pattern between groups of participants, in agreement with the in/outgroup position. From the perceiver's ingroup membership (independent of the specific race of the participant), P200 appears to be a marker of outgroup processing, and N200 appears to be a marker of ingroup processing, suggesting a differentiation of targets on the basis of ingroup and outgroup status.

Our study confirms the role of membership position in the reversion of ERP patterns. In addition, we find a correspondence between the degree of ingroup bias (IAT results) and LPP amplitude lateralization: a more racial bias effect and a stronger LPP lateralization based on compatible/incompatible blocks. Those results suggest that the physical features of faces and the semantic valence association per se do not affect the results, but that their effect is manifested in the dependence of the relation between the stimulus and the perceiver's ingroup membership. In brief, our results suggest a frontal LPP elicited by contextual blending of evaluative judgments of in-/outgroup information and positive vs. negative valence association, confirming previous research of in-/outgroup ERP modulation and frontal LPP.

### Relationship between ERPs and behavioral measures

Association rules for correlations between IAT scores and ERP data account for consistent relationships between face-IAT scores and ERPs elicited in the IAT blocks that require more effort (those that impose an association incongruent with ingroup favoritism). In other words, IAT scores computed from the reaction times of face stimuli had a strong relationship with ERP data in the most demanding task. This correlation was especially strong for ERPs generated to face stimuli. However, a relationship between VPPs elicited in response to words and IAT scores was also detected.

In ERP-questionnaire correlations, it was found that ERP data correlated most frequently with explicit scores involving the outgroup. As previously found, face ERP data are the most relevant for these associations, with peak being the most frequently correlated feature in all ERPs.

## Conclusion

To the extent of our knowledge, the present study is the first that presents late IAT brain correlates. Consistent with IAT behavioral measures, we found a frontal LPP discrimination of semantic content based on compatible blocks with prejudice against indigenous targets in the word stimulus condition only in indigenous participants, expanding the lateralization results previously found with positive-negative words [43]. This LPP showed the frontal distribution that had been previously found in evaluative categorization. In addition to finding sensitivity of the LPP component to arousal and valence cues, we found an effect of contextual blending. As an extension of N170/VPP results (Ibáñez A, Hurtado E, González R, Haye A, Manes F: Early neural markers of Implicit Attitudes: N170 modulated by intergroup and evaluative contexts in IAT, Submitted), this suggests an early and late face-word contextual blending based on the processing of word and race stimuli. In summary, our results suggest a frontal lateralization of evaluations based on the complex association between valence and membership that is relevant for ingroup/outgroup ERP research and that is consistent with implicit and explicit measures. ERP results were not inconsistent with behavioral data from our subjects that showed evidence of ingroup favoritism, which was stronger in the minority test subjects (Indigenous) than the majority (Non-Indigenous). In addition, we used a multilevel analysis and a data-mining technique in order to show a convergence of measures and the influence of ingroup favoritism at different levels of analysis. Convergent evidence was found of an association between implicit and explicit bias in our Non-Indigenous group and of a dissociation of the same in our Indigenous group.

### Data convergence

The different measures provided very interesting convergent evidence. In both groups, the explicit questionnaire, IAT behavioral measures, ERPs and their associations are all consistent in evidencing a marked predominance of ingroup distinction against outgroup, especially in the case of ingroup favoritism. In both groups, the explicit questionnaire not only shows a predominance of positive valence scores but also evidences valence differences only when subjects were asked about the ingroup and not the outgroup. In the IAT, the highest accuracies are found in the task consistent with ingroup favoritism. Correlations between the explicit questionnaire and the IAT show that the highest consistency is present for the positive explicit evaluation of the ingroup. The LPP discriminates positive stimuli in the left frontal region. Also, IAT-ERP correlations are consistent with a higher cognitive demand in the task incongruent with ingroup favoritism. These convergent results achieved by comparing different measures suggest that semantic valence associations have particular influence when the distinction between the ingroup and outgroup is at stake.

The pattern of convergence across measures was also shown to be different between the two groups. For instance, indigenous participants gave higher valence scores in the explicit measure than the non-indigenous; at the same time, only the former significantly differentiated between positive and negative valence. In the implicit measure, the general and the word-IAT scores reveal an ingroup bias only in indigenous participants. The LPP in the right frontal region elicited by words distinguishes compatibility categories with prejudice toward a minority only in the indigenous participants. Finally, there is increased association between explicit measures and ERPs when indigenous individuals evaluate unfavorable attributes of their ethnic group (positive outgroup attributes). In short, various measures converge in showing more ingroup bias in non-indigenous than in indigenous participants, in terms of both explicit and implicit measures, in associations between such measures, and in ERPs.

### Relevance for Social Cognition Research on Implicit and Explicit Measures

Electrophysiological correlates of implicit measures of prejudice are reported in the present work. This is particularly important for social psychology, since it has implications for research on attitudes in general and on prejudice toward minority groups in particular. Specifically, we focus on late electrophysiological correlates of the task involved in the IAT. The LPP results show that later electrophysiological reactions may be coherent with implicit measures of attitudes, since, in indigenous participants, higher amplitudes that are related to stimuli (words) in an unfavorable context were observed for the ingroup. The results of associations between ERPs and IAT also show more consistency in tasks unfavorable to the ingroup. Such results suggest that a crucial aspect of the IAT cognitive task lies in processing (relationships of) stimuli that are contrary to a positive representation of the target social group. The results also suggest a relatively systematic pattern in relation to explicit measures of attitude. On one hand, associations between explicit and electrophysiological measures were observed only when the target's social category was non-indigenous participants, and only concerning positive attitudes in indigenous participants. Conversely, when the target category was an indigenous ethnic group, or when the task concerned positive attitudes, dissociation between explicit and electrophysiological measures was observed. On the other hand, the relationships among explicit and implicit measures were shown to be relatively strong under certain conditions and weaker under other conditions. In particular, for non-indigenous participants, implicit prejudice was associated with a negative explicit evaluation of the indigenous and inversely associated with a positive explicit evaluation of the outgroup. But for indigenous participants, implicit prejudice was only associated with positive explicit evaluation of the ingroup; other associations were much weaker. Much needs to be done in further research to clarify these relationships; however, the general picture suggests that explicit attitude measures may be related to electrophysiological and implicit indicators, though not in a simple way. Only under some specific conditions are these coherent with explicit judgments, suggesting that different phases of behavior may be involved in a single process. In most other conditions, electrophysiological and implicit indicators seem to disclose aspects or moments of complex processing different from those involved in explicit judgments. These interpretations are consistent with results concerning the contextual malleability of implicit attitudes [53-55,61], suggesting that IAT scores may be strongly influenced by the context of IAT responses [90,91]. According to this idea, attitudes in general and intergroup attitudes in particular are to be viewed as context-dependent knowledge structures.

Some recent studies give clear evidence that this flexibility of attitudes is not restricted to explicit measures. For instance, [22] primed men and women with dual, Turkish-German national identities who were asked about positive aspects of either their German or their Turkish identity. Later, attitudes toward Germans and Turks were assessed using a special implicit association test. The result of the IAT showed that attitudes toward Turks were generally more positive than attitudes toward Germans, and that the identity priming affected men's but not women's implicit attitude scores. This implies that attitudes might be better understood if they are conceptualized not as context-independent associations stored in memory but, consistent with the notion of attitudes as temporary constructs [92], as dynamic and flexible processes of behavior construction. In the construction of behavior across time and at different scales, complementary sub-processes may be involved, from neural micro-changes to verbal expression, all of which may be eventually mediated by affective responses and cognitive evaluations. These processes have most often been studied and measured as separate phenomena, such as electric brain changes, implicit associations, and explicit judgments. On the contrary, the theoretical and methodological challenge that we would like to derive from our research is to successfully delineate the ways in which these sub-processes interact in order to explain the dynamic organization and production of behavior moment to moment.

## Authors' contributions

The study was conceived and designed by AH and AI. Data were captured and analyzed by AI, RG and EH. EH, AH, RG and AI participated in discussions about analysis and interpretation of data and wrote parts of the article. FM revised the manuscript, critically contributing to its generation. All authors read and approved the final manuscript.

## Supplementary Material

Additional file 1 **Supplementary data**. Details about validation processes, methodology and data-mining results. Click here for file
