# Supplementary material for: Contextual blending of ingroup/outgroup face stimuli and word valence: LPP modulation and convergence of measures
Source: BMC Neurosci. 2009 Jun 26;10:69. doi: 10.1186/1471-2202-10-69 (PMC2711957; doi:10.1186/1471-2202-10-69)
Supplement: Additional file 1 — Supplementary data. Details about validation processes, methodology and data-mining results. [file 1471-2202-10-69-S1.pdf]

Supplementary data:
Contextual blending of ingroup/outgroup face stimuli and word valence: LPP modulation and convergence of measures

Esteban Hurtado[1,4], Andrés Haye[5], Ramiro González[1,5], Facundo Manes[3]and Agustín Ibáñez*[1,2,3]

[1]Laboratory of Cognitive Neuroscience, Universidad Diego Portales, Santiago, Chile
[2]Researcher Career, National Scientific and Technical Research Council (CONICET), Buenos Aires, Argentina
[3]Laboratory of Experimental Psychology and Neuroscience, Institute of Cognitive Neurology (INECO), Buenos Aires, Argentina
[4]School of Engineering, Pontificia Universidad Católica de Chile, Santiago, Chile
[5]School of Psychology, Pontificia Universidad Católica de Chile, Santiago, Chile

Email: Agustín Ibañez*agustin.ibanez@udp.cl;

*Corresponding author

Participants

Indigenous origin was determined by participants identifying themselves with the Mapuche ethnic group, and by having a Mapuche last name. The nonindigenous participants were people that did not identify themselves with any Chilean ethnic group and did not come from indigenous families. Age differences were controlled in both groups ($t = 0.4487$, $gl = 31.088$, $p = 0.6567$).
All the subjects signed a consent accepting their participation and protection of their identity in accordance with the Helsinki Declaration. All the participants were right-handed, without any visual alterations or psychopathology. The nonindigenous participants were students and university professors who participated free of charge, while the indigenous participants were contacted through an indigenous community in Santiago de Chile, and they were paid for collaborating.

IAT

Validation of Linguistic Stimuli

In order to validate word content a questionnaire was used to ask how pleasant or unpleasant was a list of 150 words with a moderate frequency of use selected by using the Lifcach software. 50 psychology students participated, the average age was 19.62 ($sd = 3.33$), 67.3% were female. The participants had to answer using a Likert scale where 1 represented very pleasant and 7 very unpleasant.

An ANOVA of repeated measures was used to contrast the categorizations for the list of pleasant and unpleasant words. Important differences were obtained for the categorization of both lists $F(1, 73) = 25161$, $p < 0.0001$. From the list of pleasant words, only those that were ranked between 1 and 3 were chosen (72 pleasant words were chosen, 3 rejected), from the list of unpleasant words only those rated between 5 and 7 (71 unpleasant words were chosen, 4 rejected).

Then a validation questionnaire was used in order to rule out possible associations a priori between chosen words and the indigenous or nonindigenous ethnic groups. This questionnaire was answered by 50 psychology students; the average age was 19.32 years, 20% male. The participants used a 7 point scale, rating the association of a word with an ethnic group, 1 being an extreme association with indigenous ethnic group and 7, an extreme association with nonindigenous ethnic group. Words were evaluated as neutral with respect to the ethnic group with an average score of 4.21 ($sd = 0.5$). There was no significant and constant correlation or association found in our sample of participants between a specific certain word and one of the ethnic groups.

Nevertheless, as a protective measure, those words that on average were ranked below the 5th percentile (3.40; tendency to associate with indigenous group) or over the 95th percentile (5.03; tendency to associate with nonindigenous group) were not included. Thus, from the 143 words proposed, 9 were eliminated because they could be associated with indigenous people and 8 because they could be associated with nonindigenous people. As a result of these two preliminary studies, 126 words were used in the final research.

Validation of Pictorial Stimuli

The nonindigenous photographs were of students and professors, while the indigenous ones were taken at an indigenous community close to the city of Santiago de Chile. All the participants signed an informed consent accepting that their photographs would be used for experimental purposes. The photographs were standardized based on size, brightness and intensity. Only the eyes, nose and mouth zone was selected, in order to eliminate any possible distractions. A total of 324 photographs were taken, 159 of nonindigenous individuals and 165 indigenous individuals. 107 volunteers, all university students, their average age was 19.32 years, 65.7% female answered a questionnaire to validate the photographs previously obtained. On a 7 point scale the ethnic group of face was assessed, where 1 was completely indigenous, and 7 completely nonindigenous; additionally they had to answer from 1 to 7 how pleasant (1) or unpleasant (7) they found the photograph. They were asked to rate the emotion of the face between 1.- Neutral, 2.- Happy, 3.-

Afraid, 4.- Angry.

The averages obtained for the previous items tended toward the center of the scale: ethnic group of face: 4.44 ($sd = 1.99$); pleasant: 4.27 ($sd = 1.59$); and emotion: 2.34 ($sd = 1.10$). The images rated below the 5th percentile (very pleasant) or over the 95th percentile (very unpleasant) were eliminated; 87.04% of faces showing neutral emotion were rated in the nonindigenous and 80.70% in the indigenous group. These analyses helped to select the images more commonly identified with each category (indigenous or nonindigenous ethnic group), as well as images that were not affected by extreme values of pleasant or unpleasant and that represent a neutral emotion rated with an absolute percentage. The images that would be included in the final set for the IAT study and the electrophysiological study were chosen from this set of images.

The following figure shows anonimized examples of pictures that were used in the IAT.

IAT blocks and score computing procedure

In block 1, participants rated faces corresponding to indigenous and nonindigenous categories. In block 2, participants rated words as pleasant or unpleasant. Block 3, mixed categories of blocks 1 and 2, in a single task of combined classification, stimuli were rated as Indigenous–Unpleasant and Nonindigenous–Pleasant. This discrimination task was carried out in block 3 and repeated with more stimuli in block 4. In block 5, words were rated, but this time under reversed conditions, i.e., categories were in opposite top corners compared to previous blocks. In block 6, faces were also rated with the categories reversed. After applying reversed trial conditions, combined classification task followed in blocks 7 and 8, specifically rating four

types of stimuli, Nonindigenous–Unpleasant and Indigenous–Pleasant. Block 7 was a practice block, whereas block 8 was the corresponding test block.

Hence, this study involved 8 blocks for the IAT test instead of the 7 used in the original procedure [1] by Greenwald et al. The original procedure omitted the second word practice, block 5 in this study, probably because it is the same as block 1. This block was included in order to make both conditions (compatible and incompatible) similar, when implementing the electroencephalographic recording, because here the eight blocks are repeated in order to counterbalance the assignment of hands to categories, producing a total of 16 blocks. This counterbalance implied that all word practices were performed in all possible right and left-hand combinations.

The following steps describe the application of the algorithm proposed in [2] to the IAT test results relevant to this study.

1. All the data for the compatible (3 & 4) and incompatible blocks (7 & 8) was obtained from both key assignments.

2. Trails with a response time over 10000 ms were eliminated.

3. For each of the four blocks, the reaction time average for trials with a correct response was obtained.

4. A pooled standard deviation was calculated for all block 3 and 7 trials, and then another for blocks 4 and 8.

5. For each trial with an incorrect response, the reaction time was replaced by the corresponding block average (counted in step 3) plus 600 ms. This corresponds to a penalty applied to wrong responses.

6. Based on the resulting reaction times, both correct and incorrect trials, the average reaction time was calculated for each of the four blocks.

7. The average difference was calculated between block 7 and 3, and another difference between block 8 and 4.

8. Each average was divided by the corresponding pooled standard deviation (counted in step 4).

9. The IAT score is the average of the two values obtained in the previous step.

Questionnaire

As a result of removing questionnaire items from the study until obtaining Cronbach's alpha coefficients higher than 0.7, the internal consistency rates were as follows.

Participants	Target stimuli	Valence	Final item count	Final Cronbach's alpha
Non-indigenous	Non-indigenous	Positive	16	0.7358
Non-indigenous	Indigenous	Positive	20	0.7165
Non-indigenous	Non-indigenous	Negative	16	0.7651
Non-indigenous	Indigenous	Negative	18	0.7229
Indigenous	Non-indigenous	Positive	21	0.7935
Indigenous	Indigenous	Positive	12	0.7251
Indigenous	Non-indigenous	Negative	20	0.7279
Indigenous	Indigenous	Negative	25	0.8436

ERP recording

Hardware and software platform

E-prime software was used to design the paradigm program and presentation screens. Signals were recorded on-line using a GES300, 129-channel system with HydroCel Sensors from Electrical Geodesic Inc (see Figure 2) with a DC coupling Amplifier, 24-bit A/D converter, 200 MΩ input impedance, 0.7 μV RMS/1.4 μV pp noise; and the NetStationTM software.

Statistical analyses

IAT Test and Explicit Questionnaire

The three implicit racial bias rates produced by the IAT test (accuracy and RTs of faces, words, and combined) together with the explicit rate obtained for each question block in the questionnaire were the behavioral response variables taken into consideration.

The relevant factors in the IAT test were: experimental group (indigenous/nonindigenous) and type of stimulus in calculations (faces/words). Therefore, the results were grouped in four measurement categories. For each category the hypothesis that the measures were similar to zero was verified, therefore racial bias would not be detected. Welch's t-tests were used on two samples and two tails. The resulting four p-values were adjusted by the Holm-Bonferroni correction, in order to control the possibility of an I-type error. Therefore, a criterion was used to determine which experimental groups presented racial bias based on the IAT test and what type of stimulus proved this effect.

As for the explicit instrument, the factors involved were the participant group (indigenous or nonindigenous), the Target's social category which the questions included refer to (indigenous or

nonindigenous) and the attribute valence (positive or negative). A variance analysis (ANOVA) was applied to explore the tendencies in the results, using a mixed model that had each subject as a random effect linked to the three factors. For significant effects and interactions ($\alpha = 0.05$), averages and contrasts were calculated with Tukey's post-hoc tests.

The relationship between measures obtained from the IAT test and the questionnaire was evaluated, calculating lineal correlations, in order to explore which aspects were consistent between both types of measure. Taking into account the IAT scores for faces, words and general, together with the questionnaire scores from the questions with positive or negative valence about indigenous or nonindigenous groups, the result consisted of (for each participant group) three IAT measures and four explicit measures, producing twelve correlations in each case.

ERPs

For each component a mixed ANOVA of repeated measures with 4 within-subject factors was performed: Stimuli (WORD vs. FACE); Valence association (POSITIVE vs. NEGATIVE); Social category association (stimulus association to INGROUP vs. OUTGROUP context) and ROI (RA, LA, Cz, RP, LP [for LPP]) and a Participant group (INDIGENOUS vs. NONINDIGENOUS). The same analysis was carried out replacing the Social category association factor, i.e., grouping the SCA variable by Indigenous vs. nonindigenous stimuli. For the LPP, an analysis of compatible and incompatible categories with prejudice toward indigenous minority was included. Univariate comparisons were done whenever necessary. Results were corrected with the Greenhouse-Geisser and Bonferroni's methods to adjust the univariate output of repeated measures ANOVA for violations of the compound symmetry assumption. To calculate post-hoc contrasts, the LSD test was used. The average number of artifact-free trials across conditions was 84.3% for nonindigenous and 81.4% for indigenous groups.

Apriori algorithm

In order to use the algorithm, each relevant correlation was coded as a tuple that specifies the levels of the factors that define the two variables, categorical information that enabled a tabular organization of data. An item is the assignment of a level to a factor (e.g., Group = Indigenous). The support of an itemset is the number of tuples that contain it. Itemsets with more support are considered frequent sets, and it is on these sets that the method is based on in order to discover rules present in the data. In turn, a rule is a causal implication where an itemset determines another set. If X and Y are two different itemsets, the

strength of the rule is evaluated by the following confidence measure:

$$\text{confidence}(X) = \frac{\text{support}(X \cup Y)}{\text{support}(X)}$$

In order to discover the more reliable rules present in the database, the Apriori algorithm [3] uses a scheme that first searches for frequent itemsets, then it creates potential rules and finally it evaluates their reliability. All non-empty subsets of frequent itemsets s are included. The rule $a \rightarrow (s - a)$ is generated for each subset a. In principle generating a rule for each subset should be a slow procedure. However, the Apriori algorithm takes advantage of the fact that by adding an item to a set its support does not increase. Therefore, it eliminates unnecessary evaluations of rules, and results are obtained in reasonable time.

Apriori algorithm results for correlations between IAT scores and ERP features

When items to the left side of the arrow are true for a pair of correlated variables, items at the right side tend to be true. When confidence (Conf.) is 1, the rule holds true for all relevant observations. Note that N170/VPP data from a previous study [4] have been included so to enrich the range of relationships that can be found.

All association rules shown in the table have a confidence of 1. This indicates that no exception was found for any of them. Rules (1) to (4) show that a remarkable set of correlations between face IAT and ERP occurred in the incompatible task for non-indigenous subjects and in the compatible task for indigenous subjects. In other words, peak values for different ERP waves showed correlations of relevant magnitude (bigger than 0.3) with face IAT scores, in IAT trials having ingroup/negative and outgroup/positive associations. Such ERP peaks mainly occurred in response to faces (rules 11 to 15). VPP peak values that correlate well with IAT scores were always elicited in response to words (rule 5).

	Rule			Support	Confidence
1	Incompatible task<br>Peak-value feature<br>Face IAT	→	Non-indigenous participants	10	1
2	Peak-value feature<br>Non-indigenous participants<br>Face IAT	→	Incompatible task	10	1
3	Incompatible task<br>Peak-value feature<br>Face IAT	→	Indigenous participants	10	1
4	Peak-value feature<br>Indigenous participants<br>Face IAT	→	Compatible task	10	1
5	VPP feature<br>Peak-value feature	→	Word EEG stimulus	10	1
6	VP electrodes	→	VPP feature	10	1
7	Right electrodes<br>Face IAT	→	Peak-value feature	8	1
8	VP electrodes<br>Latency feature	→	VPP feature	8	1
9	Face EEG stimulus<br>VPP feature	→	Latency feature	8	1
10	Word EEG stimulus<br>Right subgroup	→	Peak-value feature	8	1
11	Word EEG stimulus<br>Incompatible task<br>Peak-value feature<br>Non-indigenous participants	→	Face IAT	7	1
12	Word EEG stimulus<br>Incompatible task<br>Peak-value feature<br>Face IAT	→	Non-indigenous participants	7	1
13	Word EEG stimulus<br>Peak-value feature<br>Non-indigenous participants<br>Face IAT	→	Incompatible task	7	1
14	Word EEG stimulus<br>Compatible task<br>Peak-value feature<br>Indigenous participants	→	Face IAT	7	1
15	Word EEG stimulus<br>Compatible task<br>Peak-value feature<br>Face IAT	→	Indigenous participants	7	1

Apriori algorithm results for correlations between explicit question scores and ERP features

When items to the left side of the arrow are true for a pair of correlated variables, items at the right side tend to be true. When confidence (Conf.) is 1, the rule holds true for all relevant observations. N170/VPP

data from a previous study [4] have been included so to enrich the range of relationships that can be found. Correlations between ERP measures and questions about the outgroup (specially of positive valence) were about the non indigenous group (rules 1, 2). More clearly, this means that in the indigenous group a set of relevant correlations was found between ERP measures and questions about the non indigenous group. This is then confirmed by rules 4 to 11. With less confidence than the previous rules, proposition (12) states that correlations between face ERP measures and explicit questions were found for questions about the non indigenous. The most frequently correlated ERP feature was peak value (rules 13 and 14).

	Rule			Support	Confidence
1	Outgroup RSP[1]	→	Non-indigenous TSC[2]	50	1
2	Positive valence<br>Outgroup RSP[1]	→	Non-indigenous TSC[2]	44	1
3	Non-indigenous participants	→	Non-indigenous TSC[2]	39	1
4	Peak-value feature<br>Outgroup RSP[1]	→	Non-indigenous TSC[2]<br>Positive valence	39	1
5	Peak-value feature<br>Non-indigenous TSC[2]<br>Outgroup RSP[1]	→	Positive valence	38	1
6	Peak-value feature<br>Positive valence<br>Outgroup RSP[1]	→	Non-indigenous TSC[2]	38	1
7	Peak-value feature<br>Outgroup RSP[1]	→	Positive valence	38	1
8	Peak-value feature<br>Outgroup RSP[1]	→	Non-indigenous TSC[2]	38	1
9	Peak-value feature<br>Non-indigenous TSC[2]	→	Positive valence	42	0.95
10	Peak-value feature<br>Positive valence	→	Non-indigenous TSC[2]	42	0.95
11	Peak-value feature<br>Non-indigenous TSC[2]<br>Positive valence	→	Outgroup RSP[1]	40	0.95
12	Facial stimulus	→	Non-indigenous TSC[2]	38	0.92
13	Peak-value feature	→	Positive valence	46	0.91
14	Peak-value feature	→	Non-indigenous TSC[2]	46	0.91
15	Peak-value feature<br>Non-indigenous TSC[2]	→	Outgroup RSP[1]	42	0.9

[1]RSP stands for *relative social position.*
[2]TSC stands for *target social category.*

References

1. Greenwald AG, McGhee DE, Schwartz JLK: **Measuring individual differences in implicit cognition: The Implicit Association Test**. *Journal of Personality and Social Psychology* 1998, (74):1464–1480.
2. Greenwald AG, Nosek BA, Banaji MR: **Understanding and using the implicit association test: I. An improved scoring algorithm.** *Journal of Personality and Social Psychology* 2003, **85**(2):197–216.

3. Agrawal R, Srikant R: **Fast Algorithms for Mining Association Rules in Large Databases**. In *VLDB'94, Proceedings of 20th International Conference on Very Large Data Bases, September 12-15, 1994, Santiago de Chile, Chile.* Edited by Bocca JB, Jarke M, Zaniolo C, Morgan Kaufmann 1994:487–499.

4. Ibáñez A, Hurtado E, González R, Haye A, Manes F: **Early neural markers of Implicit Attitudes: N170 modulated by intergroup and evaluative contexts in IAT** (Submitted).
